# Glucocorticoids Reduce Sepsis by Diminishing Dendritic Cell Responses

**DOI:** 10.1371/journal.pbio.1002270

**Published:** 2015-10-06

**Authors:** Richard Robinson

**Affiliations:** Freelance Science Writer, Sherborn, Massachusetts, United States of America

## Abstract

How does the body's immune system strike the delicate balance between under- and over-response? A new study shows that glucocorticoids limit the production of the proinflammatory cytokine interleukin-12 by dendritic cells in response to invading bacteria, thereby helping to avoid sepsis. Read the Research Article.

Inflammation is an important tool in the fight against infection, but it comes with risks, especially the exaggerated inflammatory response known as sepsis. Characterized by fever or hypothermia, a racing heart, and hyperventilation, sepsis may resolve on its own, or progress to organ dysfunction and septic shock, a major cause of mortality in critically ill patients. On the way to restoring homeostasis, the immune system may overcompensate, and the patient may develop a profound and life-threatening postseptic immunosuppression.

A common trigger for sepsis is the bacterial cell wall component lipopolysaccharide (LPS), exposure to which induces production of proinflammatory cytokines, including interleukin 12 (IL-12). Immune cells called dendritic cells help regulate inflammation, and several lines of evidence suggest a subpopulation of these (CD8^+^ dendritic cells) may be involved in the sepsis response, including the fact that they are a major source of IL-12, and that the resolution of sepsis is accompanied by a loss of these dendritic cells.

However, the mechanisms through which dendritic cells may regulate sepsis, and in turn be regulated by other immune components, are unclear. In a new study in *PLOS Biology*, Caiyi Li, Ivana Munitic, Jonathan Ashwell, and colleagues show that endogenous glucocorticoids (GCs) suppress the dendritic cell response to LPS exposure, reducing IL-12 production and promoting loss of dendritic cells (Fig 1).

**Fig 1:**
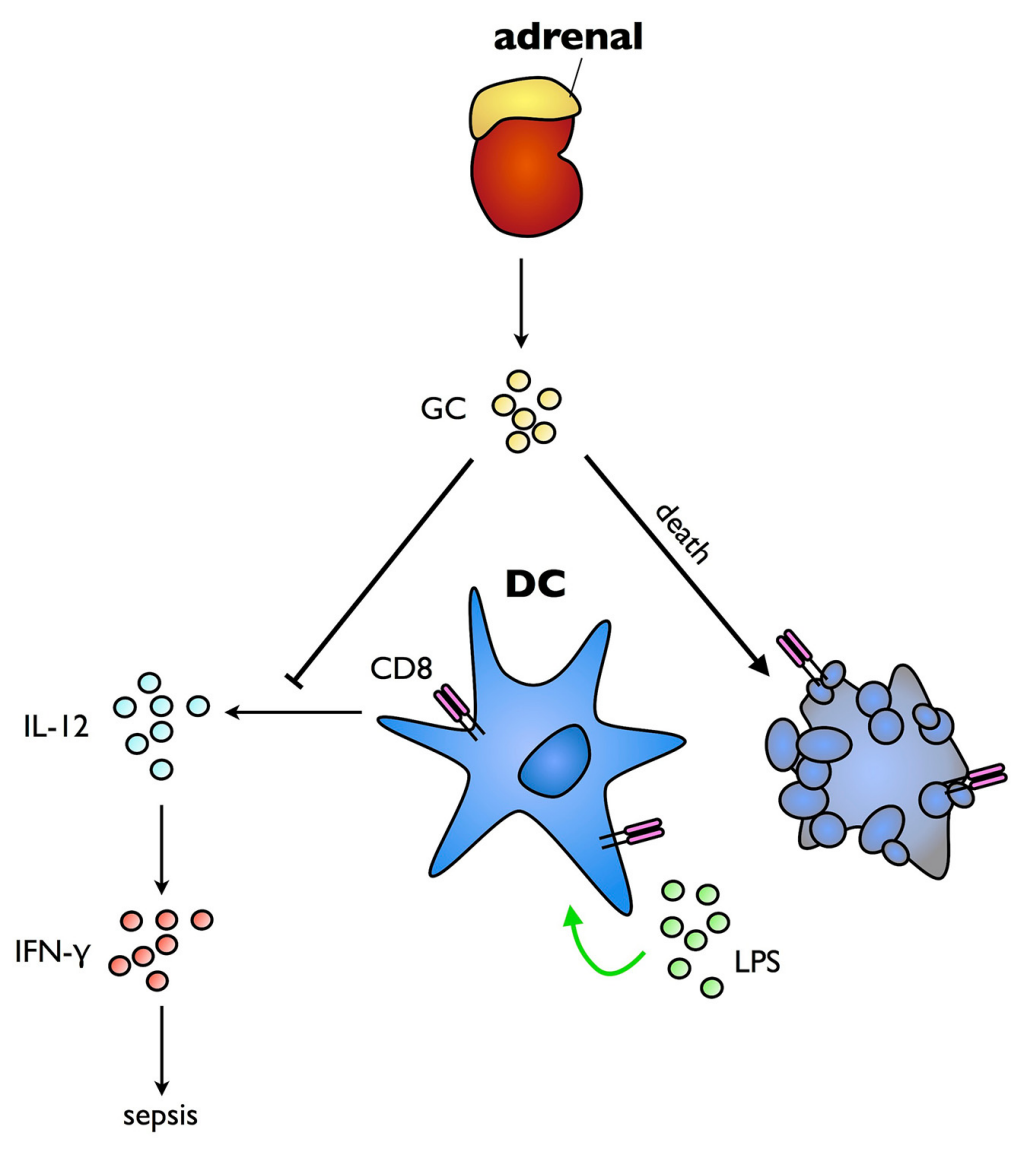
GCs act on dendritic cells to ameliorate endotoxin-induced sepsis. Endogenous GCs act as lifeguards during the hyperinflammatory phase of sepsis by suppressing IL-12 production and eliminating a major source, CD8^+^ dendritic cells. *Image credit*: *Maria Letizia Giardino Torchia*.

A role for GCs in regulation of sepsis had been suggested based on the recognition that patients with insufficient production of the hormones were at risk for prolonged sepsis. To explore the potential influence of GCs on dendritic cells, the authors created mice lacking the GC receptor only in dendritic cells. When exposed to LPS, the mice developed severe hypothermia, and most died, unlike their wild-type littermates, which had milder hypothermia and quickly recovered. Levels of IL-12 were elevated in mice without the receptor long after they had returned to normal in wild-type mice, and production of interferon-gamma, a proinflammatory cytokine downstream from IL-12, was also highly elevated, all supporting the hypothesis that a critical function of GCs are to limit the inflammatory action of dendritic cells.

The authors found they could mitigate the inflammatory response in receptor-deficient mice by injecting them with antibodies against IL-12 before exposure to LPS, demonstrating the importance of IL-12 signaling in sepsis. In cell culture, IL-12 production could be suppressed by exposure to a synthetic GC, corticosterone, but only if the GC receptor was present, confirming the role of the endogenous hormone in damping down sepsis. The authors also showed that the number of dendritic cells was increased post-sepsis in mice missing the receptor, suggesting a role for GC signaling in reducing dendritic cell numbers as part of the postsepsis response.

The same mechanisms were at work in development of LPS “tolerance,” in which a sublethal dose of LPS induces a hyporesponsiveness to a subsequent higher dose. Absence of the GC receptor prevented development of tolerance, leaving receptor-deficient mice at risk for severe hypothermia and death upon second exposure to LPS, while wild-type mice were protected.

Corticosteroids are already in use for treatment of sepsis in patients at risk, based on empiric evidence of their effectiveness. The discovery of a central role of dendritic cells in promoting sepsis is likely to promote a search for treatments focused more directly on this cell population, to replace or augment the more systemic effects of steroids. But researchers will have to design such a therapy carefully, since aggressive reduction in dendritic cell numbers or activity may lead to postsepsis immunosuppression. Further study of the mechanism of this phase of the immune response may point the way toward safer, highly targeted therapies that can prevent or reverse sepsis while maintaining a healthy immune response to new sources of infection.

## Abbreviations

GC: glucocorticoid
IL-12: interleukin 12
LPS: lipopolysaccharide
